# Development of a supportive mHealth device for persons with schizophrenia spectrum disorders (KisoLight_App_): a usability study within a participant-involvement principle

**DOI:** 10.3389/fpsyg.2025.1727156

**Published:** 2026-01-12

**Authors:** Laura Fässler, Balahan Ersöz, Inge Hahne, Sarah Koop, Kerem Böge

**Affiliations:** 1 Department of Psychiatry and Neurosciences, Charité – University Medicine Berlin, Berlin, Germany; 2 Department of Education and Psychology, Freie Universität Berlin, Berlin, Germany; 3 Department of Psychology, Brandenburg Medical School, Neuruppin, Germany; 4 German Center for Mental Health (DZPG), Partner Site Berlin/Potsdam, Berlin, Germany

**Keywords:** digital interventions, digital mental health, mHealth, psychosis, schizophrenia

## Abstract

**Background:**

To address the severe treatment gap in persons with schizophrenia spectrum disorders (SSD), this study aims to examine the usability of and satisfaction with a novel and initial mHealth application (KisoLight_App_) for SSD in Germany. The app is designed as a stand-alone tool that offers specific features such as symptom monitoring, medication adherence, and activity planning.

**Method:**

Within a single-arm usability trial, *N* = 19 participants engaged with the app over 2 weeks and completed baseline and post-engagement assessments. Quantitative and qualitative measures were included to assess feedback for the continuous development of the Kiso_App_.

**Results:**

The majority of participants engaged with the app regularly (*n* = 17 [89.50%], multiple times per week or daily). Descriptive results indicate a high usability and satisfaction with the app. Although a few unserious unwanted events (UEs) were reported, there were no serious UEs or adverse treatment reactions (ATR), indicating the safety of the app.

**Conclusion:**

The KisoLight_App_ for persons with SSD seem to be a safe, usable, and satisfying tool for supporting the addressed population. Future studies should examine an enhanced version of the app within a randomized controlled trial, including a larger sample size and additional therapeutic features.

## 1 Introduction

According to the *International Classification of Diseases* (ICD-10; Graubner and Auhuber, 2019), schizophrenia spectrum disorders (SSD), including schizophrenia and schizoaffective disorder, are defined by core symptoms such as positive symptoms (e.g., hallucinations, paranoia), negative symptoms (e.g., anhedonia, social withdrawal), and cognitive deficits. The lifetime prevalence of schizophrenia has been estimated at approximately 2.5% (Simeone et al., 2015), with annual treatment expenses ranging from 9.63 to 13.52 billion euros in Germany (Frey, 2014). Globally, SSD rank among the most debilitating diseases (Charlson et al., 2018) and high medication non-adherence in persons with SSD (Guo et al., 2023) is frequently associated with relapses. With an 80% relapse rate within 5 years, SSD often follows a chronic and severe course (Alvarez-Jimenez et al., 2012).

Since persons with SSD face severe limitations in treatment availability caused by structural deficits and limited accessibility (Jablensky, 2013; Lora et al., 2012), it is crucial to examine novel and low-threshold approaches. The emerging field of mobile health (mHealth) could contribute to an increased accessibility and might offer effective support within the addressed population. MHealth refers to the use of mobile devices, such as smartphones, tablets, or other wireless devices, to support public and medical health practices (Olla and Shimskey, 2015). In general, a variety of smartphone applications aim to address mental health problems by monitoring symptoms (Dogan et al., 2017) or delivering interventions based on psychological and psychotherapeutic principles (Rathbone et al., 2017; Pillny et al., 2025). The current literature indicates that mHealth apps are feasible, acceptable, and partially effective in treating different mental health domains and disorders (Borghouts et al., 2021; Wang et al., 2018).

Numerous mHealth applications have been tested in persons with SSD, such as devices for medication adherence monitoring, multiple forms of app-delivered therapy, skills training, or relapse prediction (Chivilgina et al., 2020). For some, the feasibility, acceptability, and safety have been demonstrated (Gumley et al., 2022). However, only a minority of accessible and appropriate apps for SSD specifically target psychosis (Kwon et al., 2022), while engagement is often low or unclearly reported (de Simões Almeida and Marques, 2023; Cipriani et al., 2025). Since most affected persons own mobile phones and show an increased interest in mHealth applications for self-management (Firth et al., 2016), it is crucial to evaluate further the potential benefits and challenges of mHealth apps for SSD.

To develop effective functions of mHealth tools that have the potential to increase engagement and their efficacy, the involvement of persons with lived experiences is a specifically essential aspect in recommendations for best-practice research (Gühne et al., 2019; Schütt et al., 2023). Within a participant-involvement approach, the quality of and adherence to mHealth applications can be improved by gaining a profound understanding of the perspective and psychosocial context of the users (Yardley et al., 2015). Previous studies showed that significant predictors for acceptance of mHealth devices are the usefulness, attributes of users, and the need for social support (Harst et al., 2019). Within a person-based app development, those main factors could lead to an increased engagement with and adherence to mHealth solutions (Torous et al., 2018). Since engagement with mHealth in SSD seems to be one of the major difficulties that is often either low or insufficiently reported (de Simões Almeida and Marques, 2023), the examination of engagement predictors need to be addressed in future research (Eisner et al., 2025; Smith et al., 2025). Besides predictors of improved usability, adverse or unwanted events (UEs) are an essential but underreported aspect of digital intervention trials (Bradstreet et al., 2019; Allan et al., 2024) that should be considered when examining mHealth approaches for SSD. This is not only essential to gain insights into adverse reactions to mHealth interventions but also to determine predictors of engagement and drop-outs (Rozental et al., 2014).

To address gaps in understanding mHealth devices for SSD and to develop tailored solutions, this usability study evaluates the KisoLight_App_, a newly developed mHealth application for SSD in Germany. The app’s first version functions as a stand-alone tool for daily support regarding, e.g., symptom monitoring and medication adherence, complemented by targeted notifications and practical features. This study focuses on user-reported feasibility and satisfaction, employing a participant-involvement approach by incorporating participants feedback in each stage to conduct an iterative, user-centered development. Additionally, we will analyze content engagement, identifying which app features users find particularly helpful. To gain a comprehensive understanding of user experiences, we will report UEs based on established frameworks (Gómez Bergin et al., 2023).

## 2 Methods

### 2.1 Study design

A single arm usability trial was conducted at the Department of Psychiatry and Psychotherapy at the Charité – University Medicine Berlin, Campus Benjamin Franklin. Data were collected within a mixed-method study design, using quantitative and qualitative outcomes. The study was approved by the ethics committee of the Charité – University Medicine Berlin (EA2/053/24).

### 2.2 Participants

The sample of *N* = 19 participants was recruited at the inpatient ward and outpatient department of the Charité – University Medicine Berlin, Campus Benjamin Franklin. The recruitment was conducted from May 2024 to December 2024. Inclusion criteria were (1) diagnosis of SSD according to ICD-10 (F2X.X; 1) by a qualified psychiatrist or psychotherapist, (2) age between 18 and 75 years, (3) written informed consent after study clarification, (4) sufficient command of the German language, and (5) the ability to use a smartphone or the willingness to learn it. Exclusion criteria were: (1) neurological disorder, (2) acute suicidality, assessed in the screening procedure and according to professional assessment (see Supplementary material A), (3) acute substance abuse or dependency, (4) acute electroconvulsive therapy, (5) no mobile phone with a sufficient Software-version (iOS: at least version 14, Android: at least version 10), and (6) item score >5 on the positive scale of the Positive and Negative Syndrome Scale (PANSS) at baseline.

### 2.3 Procedure

**Figure 1:**
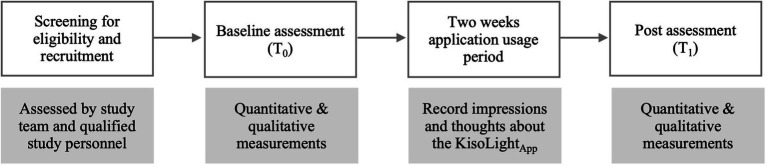
Recruitment process and study procedure. T0 = baseline assessment, T1 = post-app use assessment.

A multidisciplinary and qualified study team of psychologists and psychiatrists at the Charité – University Medicine Berlin assessed the suitability for participation. Following informed consent and subsequent inclusion, participants conducted the baseline assessment (T_0_) at the study site and were given access to the app. During the baseline assessment (T_0_), participants had the option to try the KisoLight_App_ and ask questions about its usage. They were instructed to note their impressions and thoughts about the application during the study period of two-weeks on a structured note sheet, including positive and negative aspects of the KisoLight_App_, suggestions for improvement, and technical difficulties. After 2 weeks, participants conducted the post-assessment (T_1_) and received an expense allowance of 50€ (see Figure 1).

### 2.4 The KisoLight_App_

**Figure 2:**
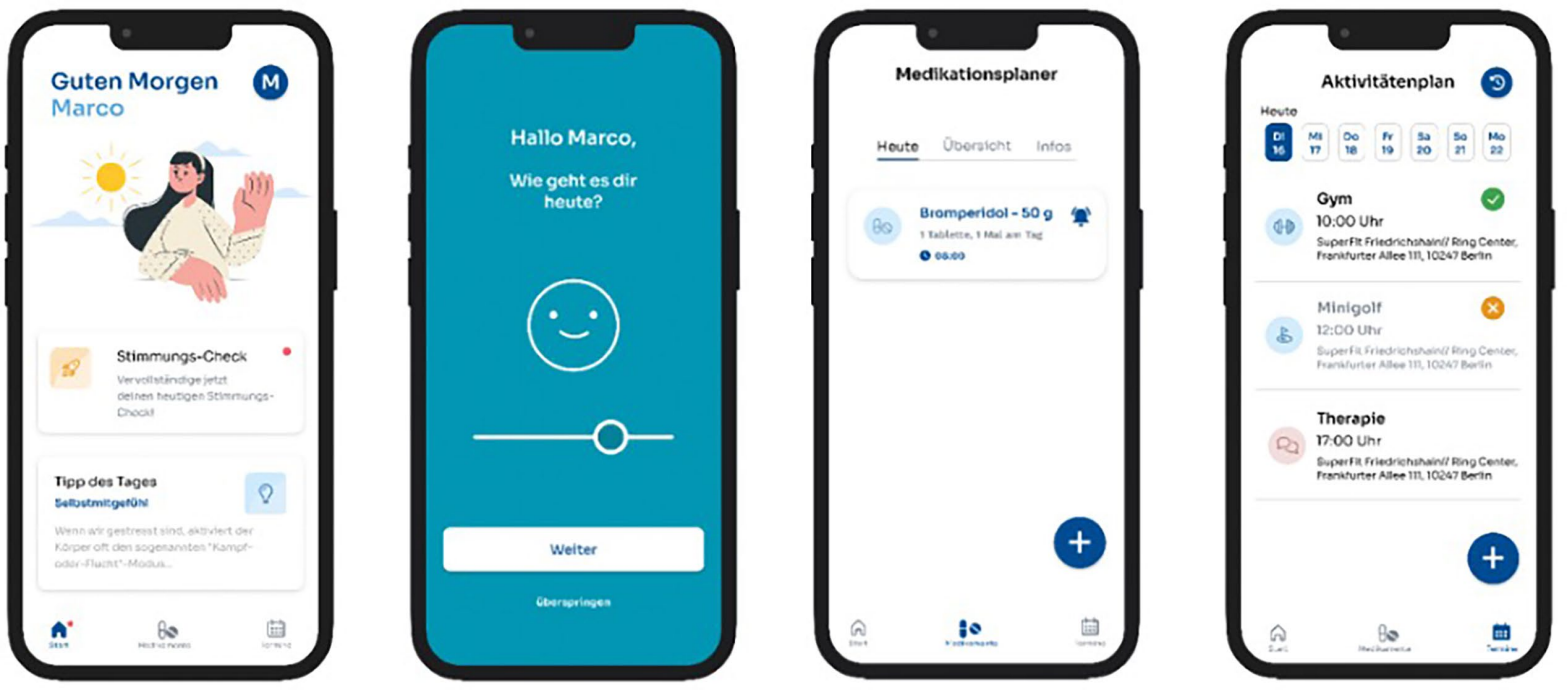
Examples for the visualization of the KisoLight_App_.

The KisoLight_App_ represents an initial version of a broader digital mental health intervention currently under development: The KisoHealth_App_ (see Figure 2). While this early version includes core functionalities such as symptom monitoring, medication adherence tracking, and an activity planner, it is deliberately limited in scope. The current version of the app is suitable for different clinical settings such as out- and in-patient care but also for different symptom severities.

#### 2.4.1 Participant-involvement approach

The app is being developed in an iterative, user-centred process. Participant involvement (via semi-structured interviews, surveys, and in-app-feedback) systematically informs feature selection, prioritization, and refinement across development stages. The present examined version of the KisoLight_App_ represents the initial form of the application, intentionally designed as a minimum viable product to allow early usability testing and iterative optimization. The app will be further developed based on the results and feedback from the current usability study. Additionally, a board of ten people with lived experiences is continuously and actively involved throughout the development process and contributes expertise. Board members are granted access to test versions of the app, which they use independently in their everyday contexts. Feedback on the current app versions is collected through semi-structured interviews, complemented by ongoing reports of technical issues and usability problems. Beyond evaluative feedback, board members are also involved in decision-making processes regarding potential improvements and the development of new features. Planned modifications and feature concepts are discussed with the board in qualitative interviews, allowing user perspectives to inform decisions on whether to implement specific design changes. This co-development approach aims to ensure that the final version of the app meets the diverse needs of its users.

#### 2.4.2 Sections and modules of the KisoLight_App_

##### 2.4.2.1 The “symptom check” section

The “symptom check” section allows participants to answer three daily questions: (1) ‘How is your current mood?’, (2) ‘How restful was your sleep last night?’ and (3) ‘How stressed are you at the moment?’. Responses are recorded on a five-point Likert scale within a slider bar. Results are graphically displayed in the app, showing individual trends regarding the three questions in the last 7 days.

##### 2.4.2.2 The “medication adherence” section

Users can set up notifications within the KisoLight_App_ that emits daily reminders for medication intake via push notifications. Users are prompted to take their prescribed medication and record the intake in the app. This feature allows for an overview of medication adherence during the past days, visualizing which doses have been taken or missed.

##### 2.4.2.3 The “activity planner” section

The “activity planner” function includes a calendar for scheduling activities and provides a list of nearby places of interest (e.g., restaurants). Users can also add personal notes related to their activities and interests.

##### 2.4.2.4 Additional features

Besides those main features, the KisoLight_App_ offers a daily advice for activities that aim to provide low threshold options to cope with daily distress such as relaxation and breathing exercises, or recommendations for relevant books, podcasts, and websites.

### 2.5 Measures

#### 2.5.1 Positive and negative symptoms

To collect data on clinical characteristics, the positive and negative scale of the Positive and Negative Syndrome Scale (PANSS) was applied to measure symptom severity of SSD (Kay et al., 1987). The PANSS is a rater-based measure that comprises seven items rated on a seven-point Likert scale (from 1 = *absent* to 7 = *extreme*) with detailed anchor descriptions. The PANSS showed good internal consistency, construct validity, and interrater reliability in previous studies (Kay et al., 1988; Peralta and Cuesta, 1994).

#### 2.5.2 Depression, anxiety, and stress

The Depression Anxiety Stress Scale (DASS-21) was used to assess symptoms of depression, anxiety, and stress with 21 items on a four-point Likert scale (Lovibond and Lovibond, 1995; Nilges and Essau, 2015). The total score ranges from zero to 63, with higher scores indicating more severe symptoms. The German version of the DASS-21 shows good reliability, construct, and structural validity (Bibi et al., 2020).

#### 2.5.3 Satisfaction

The patient’s satisfaction with the intervention was measured with the self-rated Patient Satisfaction Questionnaire (ZUF-8; Schmidt and Wittmann, 2002) which was semantically adapted for the KisoLight_App_. The scale consists of eight items that are subjectively rated on a four-point Likert scale. The ZUF-8 demonstrates internal consistency of *α* = 0.92, a high test–retest reliability and shows adequate concurrent validity with various other measures (Schmidt and Wittmann, 2002).

#### 2.5.4 Usability

The System Usability Scale (SUS) measures the usability of different websites, software, and other human-machine systems (Peres et al., 2013). It comprises 10 items rated on a five-point Likert scale, with scores ranging from zero to 100, where 100 indicates optimal usability. The SUS shows convergent validity and acceptable levels of reliability (Mol et al., 2020).

#### 2.5.5 Unwanted events

Unwanted events (UEs) were assessed by multiple measures. The Negative Effects Questionnaire (NEQ; Rozental et al., 2019) is a validated rating scale that measures negative or adverse effects of a psychological treatment. It comprises of 20 items, including two binary answer options and a five-point Likert scale. Besides, participants were asked to rate the association between a potential UEs and the KisoLight_App_ on a five-point Likert scale (1 = *Caused by the Kiso app*, 2 = *Likely caused by the Kiso app*, 3 = *Neutral*, 4 = *Likely not caused by the Kiso app*, and 5 = *Not caused by the Kiso app*) according to the UEs-checklist by Linden (2013). The NEQ is reported to have good psychometric properties (Rozental et al., 2019). Participants were instructed to report UEs that were potentially associated to the KisoLight_App_. We differentiated between UEs and Adverse Treatment Reactions (ATR), and UEs that were unrelated or related to treatment according to Linden (2013).

#### 2.5.6 Further questions

Due to the lack of tailored and validated rating scales for mHealth applications in SSD, a self-developed questionnaire (see Supplementary Table S5) was applied to evaluate specific aspects of the KisoLight_App_. Responses were measured on a five-point Likert scale (1 = *negative*, 2 = *rather negative*, 3 = *neutral*, 4 = *rather positive*, and 5 = *positive*). The applied questions were developed within a multi-disciplinary team at the Charité, consisting of clinical psychologists and physicians specialized in the field of SSD. Furthermore, software and product developers, who were involved in the development of the KisoLight_App_, contributed expertise. The questionnaire allowed for an additional user evaluation of all specific functions and potentials UEs of the KisoLight_App_. Besides, it assessed the subjectively reported engagement with the app, using questions for specific app features (see Supplementary Table S5) and one general question on app engagement (‘*Please rate how often you used the app in general*’, 1 = *not at all*, 2 = *less than once per week*, 3 = *once per week*, 4 = *multiple times per week*, 5 = *daily*). Due to reasons of data protection, we did not implement objective indicators such as screen-time or in-app event tracking.

#### 2.5.7 Participants feedback

Participants provided feedback on the KisoLight_App_ in the form of a short semi-structured interview at T_1_ (see Supplementary material B). The qualitative interview was used to include opinions of people with lived experiences in the iterative development phase for the full KisoHealth_App_. It comprised questions about general experiences with and specific features of the app to include participants in each process stage that will help to further develop the tool and its content. See Supplementary Table S1 for all measurement time points.

### 2.6 Statistical analysis

We computed descriptive statistics to evaluate demographic and clinical characteristics, as well as the satisfaction with the KisoLight_App_. Data analyses were performed using SPSS Statistics, version 27 for Windows on a per-protocol principle, only including participants with baseline and post-assessment data since the aim of the study was explicitly to gather reports about app usage and satisfaction. Therefore, we did not impute missing data. Qualitative data were included to review participants’ feedback. We analyzed and coded key themes according to the semi-structured interview guide that addresses different aspects of the KisoLight_App_ and its evaluation. Quotes will be selectively reported as examples and serve to develop the app further within an iterative, user-centered process with UX/UI Designers and are not systematically analyzed in the present paper.

## 3 Results

### 3.1 Participants

**Figure 3:**
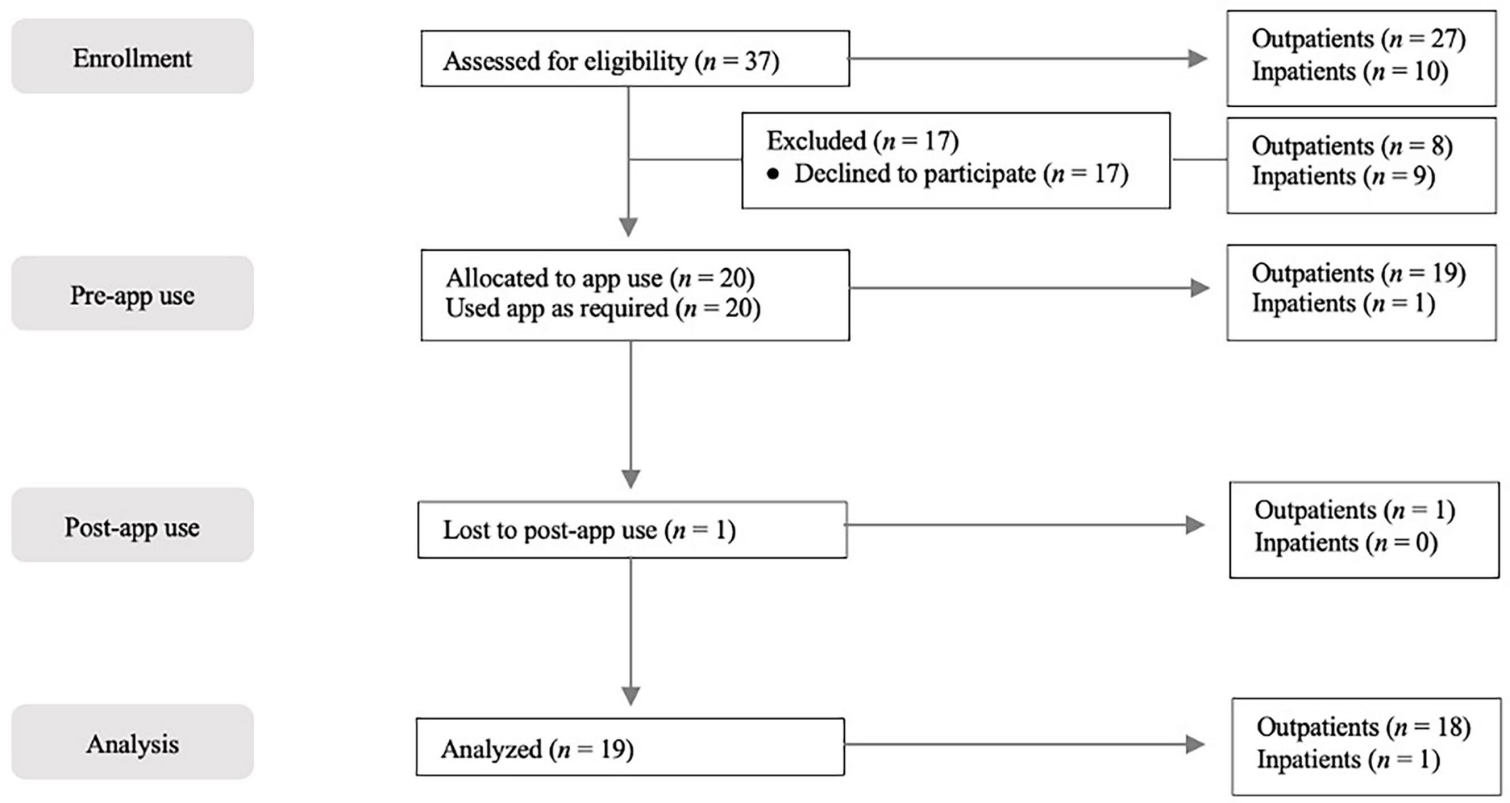
Flow diagram of the recruitment process throughout the course of the study.

A total of 20 participants were included in the study and completed the baseline assessment. One person dropped out and was no longer reachable (see Supplementary Table S6 for baseline characteristics of the whole sample). Therefore, 19 participants completed baseline and post-assessments and used the app for 2 weeks. See Figure 3 for a detailed overview about the recruitment process.

### 3.2 Demographic and baseline characteristics

The sample consisted of 19 participants with an average age of 38.4 years (*SD* = 8.86). The majority of participants identified as male (68.4%, *n* = 13), while 31.6% (*n* = 6) identified as female. Regarding primary diagnoses, most participants were diagnosed with schizophrenia (89.5%, *n* = 17), while 10.5% (*n* = 2) had a schizoaffective disorder. See Table 1 for a detailed description of demographic and clinical characteristics at baseline.

**Table 1:** Participants clinical and baseline characteristics.

Characteristics	*M* or *n*	*SD* or %
Sociodemographic data and diagnoses		
Age	38.4	8.86
Gender		
Female	6	31.6%
Male	13	68.4%
Other	–	–
Primary diagnosis		
Schizophrenia	17	89.5%
Schizoaffective disorder	2	10.5%
Comorbid diagnoses		
Depression	3	15.7%
Anxiety disorder	1	5.3%
Clinical characteristics		
PANSS		
Positive scale	14.31	5.88
Negative scale	16.42	7.11
DASS		
Depression scale	9.68	7.52
Anxiety scale	8.84	3.48
Stress scale	12.32	9.27
Total score	30.84	17.78

M = mean, SD = standard deviation, n = sample, % = percentage, PANSS = Positive and Negative Syndrome Scale, DASS = Depression Anxiety Stress Scale.

### 3.3 Primary analyses: outcomes of the subjective satisfaction with and usability of the KisoLight_App_

#### 3.3.1 Patient Satisfaction Questionnaire (ZUF-8)

**Figure 4:**
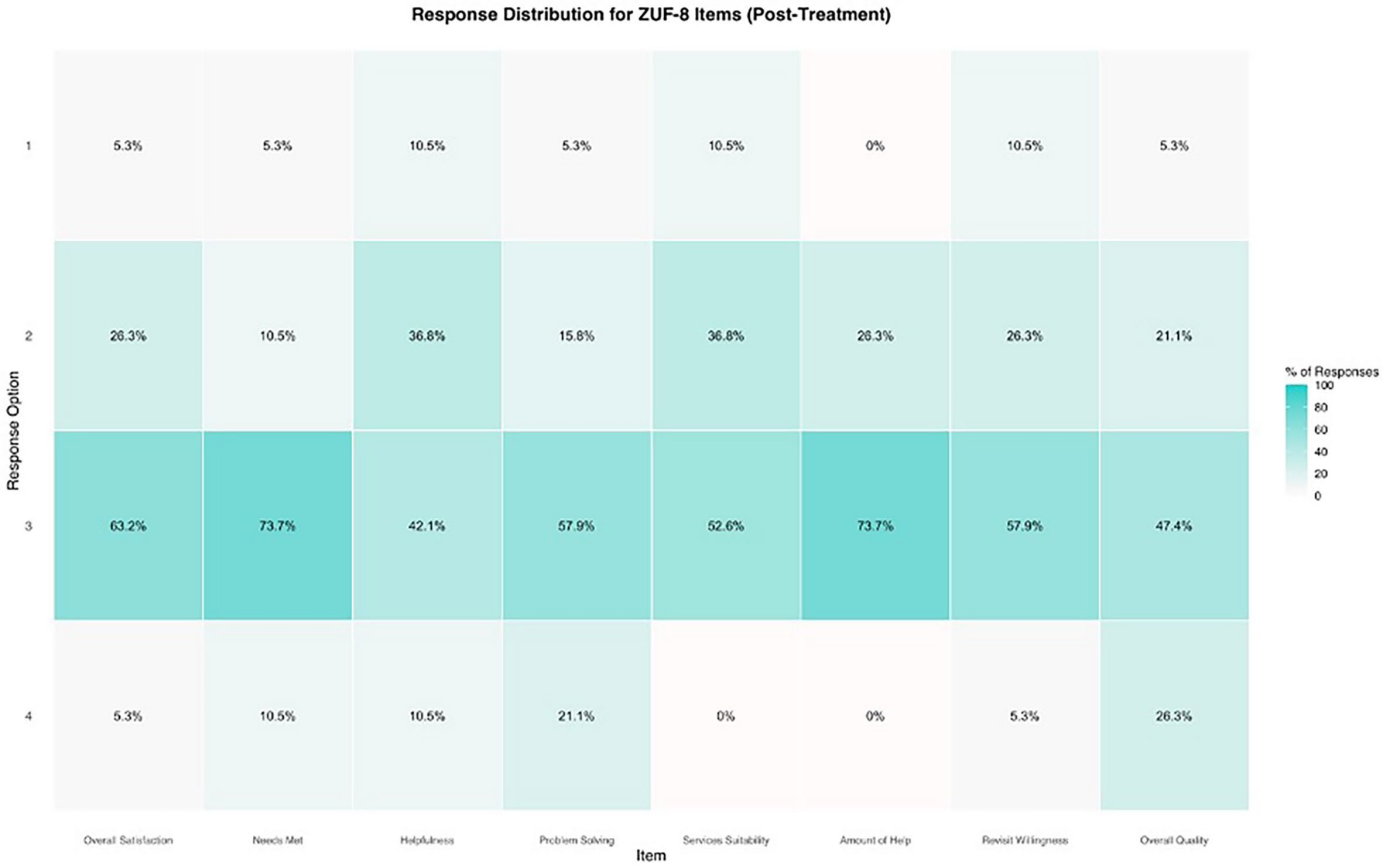
Distribution of responses regarding the subjective satisfaction (ZUF-8) with the KisoLight_App_. % = percentage. The ZUF-8 is ranging from 1 to 4, with higher scores indicating higher satisfaction.

In total, the satisfaction with the app was high. The expectations of most participants were fulfilled (*n* = 16, 84.20%) and the majority would recommend the app to a friend with similar problems (*n* = 15, 79.00%). However, only 52.60% (*n* = 10) reported that the app met the individual needs and were satisfied with the extent of help they got through the KisoLight_App_ (see Figure 4). See Supplementary Table S3 for all outcomes on satisfaction.

#### 3.3.2 System Usability Scale (SUS)

**Figure 5:**
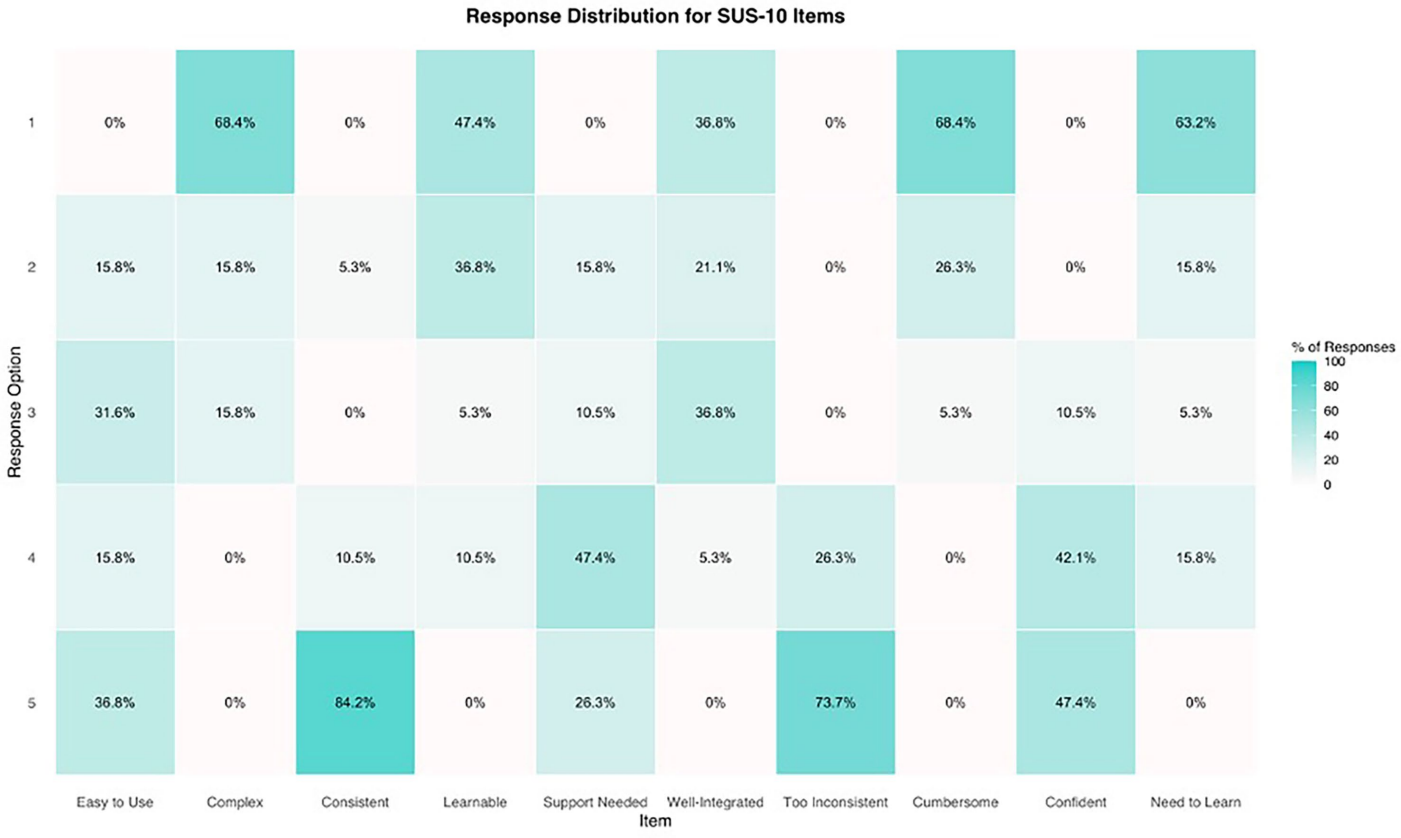
Distribution of responses regarding the system usability (SUS) of the KisoLight_App_. % = percentage. The SUS is ranging from 1 to 5, with higher scores indicating higher satisfaction.

The values of the system usability indicate high rates of easy and understandable usability. Especially, learning how to use the app was rated as very easy and quick (100%). Around half of the participants (*n* = 9, 47.40%) would use the examined version of the app, which did not include any interventional aspects, on a regular basis (see Figure 5). See Supplementary Table S4 for all outcomes on the SUS.

### 3.4 Engagement, feedback, and negative effects of the KisoLight_App_

#### 3.4.1 Engagement and feedback

A total of 19 participants used the app within the two-week intervention phase. One person dropped out and did not engage with the app. A total of eleven participants (57.9%) used the app daily, six participants used it multiple times per week (31.6%), and two participants used it once per week (10.5%). Especially the *mood check* was used frequently (*n* = 9 ‘multiple times per week’, *n* = 8 ‘daily’). Feedback gathered by qualitative interviews revealed that 18 participants (95.0%) found the app to be generally helpful. One participant stated for example ‘*Above all, the medication reminder helped me a lot because I always have trouble with my short-term memory, and therefore it is very practical.*’ and another one reported ‘*I think [the app] is very helpful, especially in crisis situations because when you slip, it’s a gradual process. (…)*’.

Feedback also revealed that 18 (95.0%) participants got anything meaningful out of the app, with one person stating ‘*I think these mood tips are really great. They make you, how do you say, more self-aware*’, or another one saying ‘*I was always happy to read something new about how to handle the day. Yes, that made me happy*’. However, 14 participants (74%) experienced any kind of technical issues, such as ‘*Sometimes the link for the daily tip did not work*’.

Detailed experiences with the KisoLight_App_ were examined with the self-developed questionnaire. It showed (1) moderate to high usefulness of the app (*n* = 12, 63.20%), (2) very high understandability (*n* = 18, 94.70%), (3) high usability (*n* = 16, 84.20%), and (4) moderate to high satisfaction with the design (*n* = 13, 68.50%).

#### 3.4.2 Negative effects (NEQ) for UEs

No serious UEs or ATR were reported during or after the study participation, neither by participants nor by the study personnel. Some reported UEs during the two-week intervention phase, whereby only a few experiences were *probably caused by the KisoLight_App_*. One participant (5.3%) reported experiencing more unpleasant feelings, which were *likely caused by the app*. Three participants (15.8%) stated that they did not always understand the app. One participant (5.3%) expressed a general lack of trust in the app. Additionally, for four participants (21.1%) the app did not provide any benefits. Five participants (26.3%) indicated that their expectations for the app were not met, with only one person tracing it to the app, and two participants (10.5%) perceived the app as demotivating. See Supplementary Table S2 for all outcomes on the NEQ.

## 4 Discussion

The evolving field of mHealth tools has the potential to increase the accessibility of available therapeutic approaches (Torous et al., 2025) and, therefore, to bridge equity gaps in the health care system for persons with SSD (Kuhn et al., 2024). Considering this potential, the present study aimed to examine for the first time the usability and satisfaction of the novel KisoLight_App_ for persons with SSD in Germany.

### 4.1 Engagement and satisfaction with the app

Findings demonstrated low participant attrition rates, with only one person dropping out of the study. A total of 19 participants with SSD completed all assessments, indicating a completion rate of 95%, which is in line with previous studies (e.g., Meyer et al., 2018; Eisner et al., 2019). Besides, results showed satisfying engagement with the app since 17 (89.50%) participants included in the study used the app on a regular basis (daily or multiple times/week). However, as our study was designed as a usability study, it only included one post-assessment and a short intervention period of 2 weeks. Since studies with longer follow-ups and more frequent assessments showed lower completion rates (e.g., Eisner et al., 2023; Smelror et al., 2019), future analyses of the full KisoHealth_App_ should consider aspects of engagement to keep the high completion rate, such as human support (Killikelly et al., 2017). Especially, objective measures of app usage and engagement should be implemented in future trials (Chen et al., 2019; Valentine et al., 2025). Moreover, some symptomatic and usability features were found to have an influential effect on engagement, such as symptom severity prior to app use (Price et al., 2022), technical support, participation, or initial training (de Simões Almeida and Marques, 2023). In our current study, we excluded persons with very severe positive symptoms to obtain initial data on more ‘stable’ patients. However, this led to limitations in conclusions on more acute patients. In future, fully powered trials on the further developed version of the KisoHealth_App_, the examination of engagement predictors will contribute to a better understanding of the app’s suitability for different disease stages and whether symptom severity, such as functioning, cognitive deficits, or severe positive symptoms, affects its efficacy.

In general, validated rating scales and participants’ feedback demonstrated high satisfaction with the app and its features, indicating that it is both acceptable and feasible for persons with SSD. However, only 52.6% of users stated that the app met their needs which is likely due to the limited functions and interventional aspects of the initial app version. Therefore, the KisoLight_App_ is currently enhanced with the help of a board of ten people with lived experiences not included in the usability study to facilitate a user-centred development of the KisoHealth_App_. Based on participant feedback, several core components were revised after the present usability study. The medication setup process was simplified through a broader redesign of the flow aimed at reducing cognitive and interactional demands, with the implementation of a predefined medication list serving as one concrete example. This revised flow was subsequently evaluated in an additional usability testing cycle. To address engagement, the additional daily advice for activities feature was expanded, and an extended set of evidence-based psychological interventions was included. Topic selection and content depth were continuously refined in collaboration with persons with lived experiences, ensuring relevance and perceived usefulness. The app is currently being developed in an ongoing iterative design process in which selected users act as continuous advisors and testers, while additional participants are involved in targeted usability evaluations. Besides, critical feedback on technical errors and specific app features identified in the present study allowed for an ongoing improvement of the mHealth program. Since no serious UEs or ATR were reported during or after the two-week usage phase, and only a small number of participants reported some unserious UEs that were *probably caused by the KisoLight_App_* and only one that was judged as *caused by the app*, it can be considered as safe for the addressed population. This aligns with previous analyses regarding the feasibility, acceptability, and safety of mHealth programs for persons with SSD (Gumley et al., 2022). Even though some studies exist that examined mHealth programs for SSD (e.g., Kwon et al., 2022; de Simões Almeida and Marques, 2023), there is still a lack in standardized guidelines for selecting appropriate and comparable measures to reduce heterogeneity in outcomes and, therefore, to determine reliable effect sizes (Bradway et al., 2020). This aspect should be considered in future trials. Our findings on negative effects and UEs could also play an important role in the use of future mHealth tools in SSD. Feelings of mistrust, as supported with our data, could possibly lead to a reduced engagement with the app. This should be addressed in the app’s future implementation by creating an easy-to-use tool with comprehensive user information, including data protection and medical counseling by physicians and psychologists, when augmenting the app in in- and out-patient settings. Furthermore, one participant in our sample reported experiencing increased unpleasant feelings during the 2 weeks of app use. This experience was attributed to the mood-tracking feature, whereby the person reportedly heightened awareness of negative mood states. To address this issue in future app iterations, we plan to incorporate targeted therapeutic strategies to manage mood deterioration. Such enhancements are expected to strengthen users’ self-efficacy and provide concrete tools for coping with potential declines in mood. Our findings on satisfaction and usability also showed that participants found the app to be easy to use, and reminders for medication and the tracking of mood were rated to be especially helpful in monitoring individual changes. Although some applications exist that focus on medication adherence (Kreyenbuhl et al., 2019), blended care CBT approaches (Garety et al., 2021), or symptom monitoring (Eisner et al., 2019), there is, to the best of our knowledge, no German mHealth tool for SSD that combines different interventional aspects in one app. In the enhanced version of the KisoHealthApp, we will include specific therapeutic components, such as audio, visual, and structured exercises, based on cognitive-behavioural therapy (Sitko et al., 2020), mindfulness-based interventions (Meinhart et al., 2025), and metacognitive strategies (Moritz et al., 2023). Besides, psychoeducational elements, a medication planner for adherence, and a mood tracker for symptom changes will be a part of future app versions to allow for a stand-alone tool that can augment the current in- and out-patient psychiatric and psychological care of persons with SSD. As part of a future randomized controlled trial with a pre-post assessment design, we will examine novel features of the app with a specific focus on symptom reduction and engagement patterns. This will allow for a better understanding of possible influencing effects on usage and beneficial changes in symptoms of SSD.

### 4.2 Limitations

Although this usability study yielded positive findings in satisfaction, usability, and safety of a novel mHealth application for persons with SSD, results are limited to a small sample size and post-assessments without experimental control or randomization. Therefore, further examination of an enhanced app version is needed, especially relating to an in-depth analysis of the feasibility and acceptability of the app in a larger sample. Besides, more rigorous verifications of engagement with the app will lead to more valid conclusions, for example, with an external validation of app engagement by tracking users’ screen time or responses to app notifications. A longer app usage phase (up to 12 weeks) and frequent outcome assessments are crucial to determine potential negative effects or actual engagement rates, which will be implemented in future studies examining the KisoHealth_App_. Since the first version of the KisoLight_App_ did not include any therapeutic interventions, our current study did not test for efficacy or symptom reduction. Within a pilot randomized controlled setting, future trials should examine the full app’s effects on symptoms and general psychopathology. Due to a limited availability of outcome measures for mHealth programs in SSD, we additionally used a non-validated rating scale to gather feedback for specific functions of the KisoLight_App_ that needs to be interpreted under caution. Despite these limitations, this study offers valuable insights into the potential of a novel mHealth technology that can support persons with SSD in their daily life and, perceptively, by managing distressing symptoms. To the best of our knowledge, it provides the first evaluation of an app for the addressed population in Germany that has the potential to improve the severe structural deficits in the treatment of persons with SSD. Furthermore, the trial was conducted within a participant-involvement principle which is in accordance with national guidelines (Schütt et al., 2023).

## 5 Conclusion

This usability study represents the first systematic evaluation of the supportive mHealth application KisoLight_App_ designed for persons with SSD. The results indicate that the application is appropriate, user-friendly, and safe for the target population. As the current version of the app did not include interventional components and primarily serves as an initial tool for further development, the present findings and particularly the qualitative feedback will inform subsequent adaptations and refinements of the app. Concurrently, the development team at KisoMind, in close collaboration with a board of people with lived experience, is working to expand the app’s interventional and functional features, with the aim of creating a more targeted and comprehensive digital intervention. Future randomized controlled trials will be essential to assess the feasibility, acceptability, and clinical efficacy of the enhanced KisoHealth_App_, which is expected to incorporate a broad array of evidence-based psychological interventions. This will contribute to improving the accessibility and quality of mental health care for persons with SSD.

## Data Availability

The raw data supporting the conclusions of this article will be made available by the authors, without undue reservation.
