# Supplementary material for: Development of a supportive mHealth device for persons with schizophrenia spectrum disorders (KisoLightApp): a usability study within a participant-involvement principle
Source: Front Psychol. 2026 Jan 12;16:1727156. doi: 10.3389/fpsyg.2025.1727156 (PMC12832653; doi:10.3389/fpsyg.2025.1727156)
Supplement: Supplementary file 1 [file Supplementary_file_1.docx]

**Supplemental Material**

**A: Suicidality Screening Questions**

**Screening for suicidality:**
*I am required to ask all study participants the following two questions. If you feel unable to answer, please let me know.*

In the past two weeks, have you experienced distressing thoughts involving a desire for your own death or self-harm?

□ **Yes** **□ No**

In the past week, have you thought about taking your own life?

□ **Yes** **□ No**

*→ If at least one suicidality question is answered with "Yes," structured assessment is required from a trained psychologist or psychiatrist is needed.*

# **B: Semi-Structured Interview**

This document contains the semi-structured interview conducted in the Kiso pilot study.

## Questions before app usage

### **Participant background**

- What are your goals and wishes (for your inpatient treatment / for your outpatient treatment)?
- How motivated do you feel to use the Kiso app? On a scale from 1 to 10?
- How would you define successful support through apps like Kiso?
- What are your expectations of the Kiso app?
- Do you already have experience using apps in general?
- *IF YES*: Also in the form of digital therapeutic applications?

We have reached the end of the conversation. Thank you very much for your time and openness! Your support is a valuable contribution to research. Do you have any questions or comments?

## Questions before app usage

(Important: Before the interview, review the participant’s answers on the NEQ again)

### **Participant’s mood, recently**

- How have you been feeling over the past few days?
- Two weeks ago you had (x, y, z) – how are these symptoms now? (Reference to PANSS)

### **Participant’s evaluation of Kiso**

"Technical errors should not influence the evaluation. We can address those separately now."

- Did any technical issues occur while using the app?
- How frequently did you use the Kiso app in your daily life?
- How did the support provided by Kiso help you achieve your goals?
- What did you think of the "Tip of the Day"?
- What did you think of the medication reminder?
- What did you think of the three daily questions on therapy-related topics?
- What did you think of the activity planner?
- Which features of the Kiso app did you find particularly helpful?
- Which features of the Kiso app did you find not very helpful?
- What difficulties did you experience while using the app?
- Were there any negative effects caused by the Kiso app?
- When did (x, y, z) occur?
- What exactly caused (x, y, z)?
- (*Reference to NEQ; if applicable*): You also mentioned (x, y, z) in the questionnaire.
- Could you describe (x, y, z) again?
- When did (x, y, z) occur?
- What exactly caused (x, y, z)?
- What features would you like to see in the Kiso app in the future?
- Do you have any suggestions for improving the Kiso app?

We have reached the end of the conversation. Thank you very much for your time and openness! Your support is a valuable contribution to research. Do you have any questions or comments?

**C: Tables**

**Table S1**

# *Schedule of the applied measures across each measurement time point*

| Time point | Baseline (T_0_) | Post assessment (T_1_) |
| --- | --- | --- |
| WEEK | 0 | 2 |
| Inclusion and exclusion criteria | x |  |
| Demographic and clinical characteristics | x |  |
| Baseline Questionnaire (*self-developed*) | x |  |
| Positive and negative scale of the Positive and Negative Syndrome Scale (*PANSS*) | x |  |
| Depression Anxiety Stress Scale (*DASS-21*) | x |  |
| Patient Satisfaction Questionnaire (*ZUF-8*) |  | x |
| System Usability Scale (*SUS*) | x | x |
| Negative effects questionnaire (*NEQ*) |  | x |
| Post Questionnaire (*self-developed*) |  | x |

**Table S2**

# *Negative Effects Questionnaire (NEQ)*

| Item | M or *n* | *SD* or % |
| --- | --- | --- |
| **Negative Effects Questionnaire** |  |  |
| I had bigger problems with my sleep. | 1.21 | 0.41 |
| 1. Yes | 4 | 21.1 % |
| 1. No | 15 | 78.9 % |
| It affected me to the following extent: | 3.0 | 1.41 |
| 1. Not at all | 0 | 0 % |
| 1. Somewhat | 2 | 50 % |
| 1. Moderately | 1 | 25 % |
| 1. Rather strongly | 0 | 0 % |
| 1. Very strongly | 1 | 25 % |
| The described situation was… |  |  |
| 1. Caused by the Kiso-App | 0 | 0 % |
| 1. Probably caused by the Kiso-App | 0 | 0 % |
| 1. Neutral | 0 | 0 % |
| 1. Probably not caused by the Kiso-App | 0 | 0 % |
| 1. Not caused by the Kiso-App | 4 | 100 % |
| I felt more stressed. | 1.16 | 0.37 |
| 1. Yes | 3 | 15.8 % |
| 1. No | 16 | 84.2 |
| It affected me to the following extent: | 3.67 | 0.57 % |
| 1. Not at all | 0 | 0 % |
| 1. Somewhat | 0 | 0 % |
| 1. Moderately | 1 | 33.3 % |
| 1. Rather strongly | 2 | 66.7 % |
| 1. Very strongly | 0 | 0 % |
| The described situation was… |  |  |
| 1. Caused by the Kiso-App | 0 | 0 % |
| 1. Probably caused by the Kiso-App | 0 | 0 % |
| 1. Neutral | 0 | 0 % |
| 1. Probably not caused by the Kiso-App | 0 | 0 % |
| 1. Not caused by the Kiso-App | 3 | 100 % |
| I was more afraid. | 1.05 | 0.22 |
| 1. Yes | 1 | 5.3 % |
| 1. No | 18 | 94.7 % |
| It affected me to the following extent: | 1.00 | 0 |
| 1. Not at all | 1 | 100 % |
| 1. Somewhat | 0 | 0 % |
| 1. Moderately | 0 | 0 % |
| 1. Rather strongly | 0 | 0 % |
| 1. Very strongly | 0 | 0 % |
| The described situation was… |  |  |
| 1. Caused by the Kiso-App | 0 | 0 % |
| 1. Probably caused by the Kiso-App | 0 | 0 % |
| 1. Neutral | 0 | 0 % |
| 1. Probably not caused by the Kiso-App | 0 | 0 % |
| 1. Not caused by the Kiso-App | 1 | 100 % |
| I became more restless. | 1.16 | 0.37 |
| 1. Yes | 3 | 15.8 % |
| **Table S2** (*continued*) |  |  |
| Item | M or *n* | *SD* or % |
| 1. No | 16 | 84.2 % |
| It affected me to the following extent: | 3.33 | 0.57 |
| 1. Not at all | 0 | 0 % |
| 1. Somewhat | 0 | 0 % |
| 1. Moderately | 2 | 66.7 % |
| 1. Rather strongly | 1 | 33.3 % |
| 1. Very strongly | 0 | 0 % |
| The described situation was… |  |  |
| 1. Caused by the Kiso-App | 0 | 0 % |
| 1. Probably caused by the Kiso-App | 0 | 0 % |
| 1. Neutral | 2 | 66.7 % |
| 1. Probably not caused by the Kiso-App | 0 | 0 % |
| 1. Not caused by the Kiso-App | 1 | 33.3 % |
| I felt a great hopelessness. | 1.05 | 0.22 |
| 1. Yes | 1 | 5.3 % |
| 1. No | 18 | 94.7 % |
| It affected me to the following extent: | 3 | 0 |
| 1. Not at all | 0 | 0 % |
| 1. Somewhat | 0 | 0 % |
| 1. Moderately | 1 | 100 % |
| 1. Rather strongly | 0 | 0 % |
| 1. Very strongly | 0 | 0 % |
| The described situation was… |  |  |
| 1. Caused by the Kiso-App | 0 | 0 % |
| 1. Probably caused by the Kiso-App | 0 | 0 % |
| 1. Neutral | 0 | 0 % |
| 1. Probably not caused by the Kiso-App | 0 | 0 % |
| 1. Not caused by the Kiso-App | 1 | 100 % |
| I experienced more unpleasant feelings. | 1.21 | 0.41 |
| 1. Yes | 4 | 21.1 % |
| 1. No | 15 | 78.9 % |
| It affected me to the following extent: | 3.25 | 1.50 |
| 1. Not at all | 0 | 0 % |
| 1. Somewhat | 2 | 50 % |
| 1. Moderately | 0 | 0 % |
| 1. Rather strongly | 1 | 25 % |
| 1. Very strongly | 1 | 25 % |
| The described situation was… |  |  |
| 1. Caused by the Kiso-App | 0 | 0 % |
| 1. Probably caused by the Kiso-App | 1 | 25 % |
| 1. Neutral | 1 | 25 % |
| 1. Probably not caused by the Kiso-App | 0 | 0 % |
| 1. Not caused by the Kiso-App | 2 | 50 % |
| I noticed that the problem for which I used the app got worse. | 1.11 | 0.31 |
| 1. Yes | 2 | 10.5 % |
| 1. No | 17 | 89.5 % |
| It affected me to the following extent: | 3.50 | 1.00 |
| 1. Not at all | 0 | 0 % |
| 1. Somewhat | 0 | 0 % |
| 1. Moderately | 1 | 50 % |
| **Table S2** (*continued*) |  |  |
| Item | M or *n* | *SD* or % |
| 1. Rather strongly | 1 | 50 % |
| 1. Very strongly | 0 | 0 % |
| The described situation was… |  |  |
| 1. Caused by the Kiso-App | 0 | 0 % |
| 1. Probably caused by the Kiso-App | 0 | 0 % |
| 1. Neutral | 0 | 0 % |
| 1. Probably not caused by the Kiso-App | 0 | 0 % |
| 1. Not caused by the Kiso-App | 2 | 100 % |
| I experienced that old, unsettling memories came back. | 1.16 | 0.37 |
| 1. Yes | 3 | 84.2 % |
| 1. No | 16 | 15.8 % |
| It affected me to the following extent: | 3.33 | 1.15 |
| Not at all | 0 | 0 % |
| Somewhat | 1 | 33.3 % |
| Moderately | 0 | 0 % |
| Rather strongly | 2 | 66.7 % |
| Very strongly | 0 | 0 % |
| The described situation was… |  |  |
| Caused by the Kiso-App | 0 | 0 % |
| Probably caused by the Kiso-App | 0 | 0 % |
| Neutral | 1 | 33.3 % |
| Probably not caused by the Kiso-App | 1 | 33.3 % |
| Not caused by the Kiso-App | 1 | 33.3 % |
| I became afraid that other people could know that I am using a health app. | 1.00 | 0 |
| 1. Yes | 0 | 0 % |
| 1. No | 19 | 100 % |
| I had thoughts that it would be better to not be here anymore or that I should take my life. | 1.00 | 0 |
| 1. Yes | 0 | 0 % |
| 1. No | 19 | 100 % |
| I started to feel ashamed for using a health app. | 1.00 | 0 |
| 1. Yes | 0 | 0 % |
| 1. No | 19 | 100 % |
| I stopped believing that things could get better. | 1.05 | 0.22 |
| 1. Yes | 1 | 5.3 % |
| 1. No | 18 | 94.7 % |
| It affected me to the following extent: | 4.00 | 0 |
| 1. Not at all | 0 | 0 % |
| 1. Somewhat | 0 | 0 % |
| 1. Moderately | 0 | 0 % |
| 1. Rather strongly | 1 | 100 % |
| 1. Very strongly | 0 | 0 % |
| The described situation was… |  |  |
| 1. Caused by the Kiso-App | 0 | 0 % |
| 1. Probably caused by the Kiso-App | 0 | 0 % |
| 1. Neutral | 0 | 0 % |
| 1. Probably not caused by the Kiso-App | 0 | 0 % |
| 1. Not caused by the Kiso-App | 1 | 100 % |
| **Table S2** (*continued*) |  |  |
| Item | M or *n* | *SD* or % |
| I started to believe that the problem I used the app for was not being influenced to the better. | 1.11 | 0.31 |
| 1. Yes | 2 | 10.5 % |
| 1. No | 17 | 89.5 % |
| It affected me to the following extent: | 2.50 | 2.12 |
| 1. Not at all | 1 | 50 % |
| 1. Somewhat | 0 | 0 % |
| 1. Moderately | 0 | 0 % |
| 1. Rather strongly | 1 | 50 % |
| 1. Very strongly | 0 | 0 % |
| The described situation was… |  |  |
| 1. Caused by the Kiso-App | 0 | 0 % |
| 1. Probably caused by the Kiso-App | 0 | 0 % |
| 1. Neutral | 0 | 0 % |
| 1. Probably not caused by the Kiso-App | 0 | 0 % |
| 1. Not caused by the Kiso-App | 2 | 100 % |
| I seem to have developed an addiction to the app | 1.00 | 0 |
| 1. Yes | 0 | 0 % |
| 1. No | 19 | 100 % |
| I did not always understand the app. | 1.16 | 0.37 |
| 1. Yes | 3 | 15.8 % |
| 1. No | 16 | 84.2 % |
| It affected me to the following extent: | 2.00 | 1.00 |
| 1. Not at all | 1 | 33.3 % |
| 1. Somewhat | 1 | 33.3 % |
| 1. Moderately | 1 | 33.3 % |
| 1. Rather strongly | 0 | 0 % |
| 1. Very strongly | 0 | 0 % |
| The described situation was… |  |  |
| 1. Caused by the Kiso-App | 0 | 0 % |
| 1. Probably caused by the Kiso-App | 2 | 66.7 % |
| 1. Neutral | 0 | 0 % |
| 1. Probably not caused by the Kiso-App | 0 | 0 % |
| 1. Not caused by the Kiso-App | 1 | 33.3 % |
| I had no trust in the app | 1.05 | 0.22 |
| 1. Yes | 1 | 5.3 % |
| 1. No | 18 | 94.7 % |
| It affected me to the following extent: | 3.00 | 0 |
| 1. Not at all | 0 | 0 % |
| 1. Somewhat | 0 | 0 % |
| 1. Moderately | 1 | 100 % |
| 1. Rather strongly | 0 | 0 % |
| 1. Very strongly | 0 | 0 % |
| The described situation was… |  |  |
| 1. Caused by the Kiso-App | 0 | 0 % |
| 1. Probably caused by the Kiso-App | 1 | 100 % |
| 1. Neutral | 0 | 0 % |
| 1. Probably not caused by the Kiso-App | 0 | 0 % |
| 1. Not caused by the Kiso-App | 0 | 0 % |
| I had the feeling that the app did not yield me anything. | 1.21 | 0.41 |
| **Table S2** (*continued*) |  |  |
| Item | M or *n* | *SD* or % |
| 1. Yes | 4 | 21.1 % |
| 1. No | 15 | 78.9 % |
| It affected me to the following extent: | 1.75 | 0.50 |
| 1. Not at all | 1 | 25 % |
| 1. Somewhat | 3 | 75 % |
| 1. Moderately | 0 | 0 % |
| 1. Rather strongly | 0 | 0 % |
| 1. Very strongly | 0 | 0 % |
| The described situation was… |  |  |
| 1. Caused by the Kiso-App | 0 | 0 % |
| 1. Probably caused by the Kiso-App | 3 | 75 % |
| 1. Neutral | 0 | 0 % |
| 1. Probably not caused by the Kiso-App | 0 | 0 % |
| 1. Not caused by the Kiso-App | 1 | 25 % |
| I did not have the feeling that my expectations for the app were met. | 1.26 | 0.45 |
| 1. Yes | 5 | 26.3 % |
| 1. No | 14 | 73.7 % |
| It affected me to the following extent: | 2.00 | 1.00 |
| 1. Not at all | 2 | 40 % |
| 1. Somewhat | 1 | 20 % |
| 1. Moderately | 2 | 40 % |
| 1. Rather strongly | 0 | 0 % |
| 1. Very strongly | 0 | 0 % |
| The described situation was… |  |  |
| 1. Caused by the Kiso-App | 1 | 20 % |
| 1. Probably caused by the Kiso-App | 2 | 40 % |
| 1. Neutral | 0 | 0 % |
| 1. Probably not caused by the Kiso-App | 0 | 0 % |
| 1. Not caused by the Kiso-App | 2 | 40 % |
| I thought that the app was demotivating. | 1.11 | 0.31 |
| 1. Yes | 2 | 10.5 % |
| 1. No | 17 | 89.5 % |
| It affected me to the following extent: | 2.50 | 0.70 |
| 1. Not at all | 0 | 0 % |
| 1. Somewhat | 1 | 50 % |
| 1. Moderately | 1 | 50 % |
| 1. Rather strongly | 0 | 0 % |
| 1. Very strongly | 0 | 0 % |
| The described situation was… |  |  |
| 1. Caused by the Kiso-App | 0 | 0 % |
| 1. Probably caused by the Kiso-App | 2 | 100 % |
| 1. Neutral | 0 | 0 % |
| 1. Probably not caused by the Kiso-App | 0 | 0 % |
| 1. Not caused by the Kiso-App | 0 | 0 % |

*Notes.* M = mean, SD = standard deviation, n = sample, % = percentage.

**Table S3**

# *Detailed analysis of the patient satisfaction questionnaire (ZUF-8)*

| Item, post-treatment | *M* (*SD*) or *n* | Positive Rating *n* (%) or % |
| --- | --- | --- |
| **ZUF-8** |  |  |
| **1. How do you rate the quality of the KisoMind_App_?** | 2.68 (0.67) | 13 (68.50%) |
| 1. Bad | 1 | 5.3 % |
| 1. Rather bad | 5 | 26.3 % |
| 1. Good | 12 | 63.2 % |
| 1. Excellent | 1 | 5.3 % |
| **2. Did you receive the kind of app you expected?** | 2.89 (0.65) | 16 (84.20%) |
| 1. Absolutely not | 1 | 5.3 % |
| 1. Rather not | 2 | 10.5 % |
| 1. Rather yes | 14 | 73.7 % |
| 1. Absolutely | 2 | 10.5 % |
| **3. To what extent did the KisoMind_App_ meet your needs?** | 2.53 (0.84) | 10 (52.60%) |
| 1. It did not meet my needs | 2 | 10.5 % |
| 1. It only met some of my needs | 7 | 36.8 % |
| 1. It met most of my needs | 8 | 42.1 % |
| 1. It met almost all of my needs | 2 | 10.5 % |
| **4. Would you recommend the KisoMind_App_ to a friend with similar symptoms?** | 2.95 (0.78) | 15 (79.00%) |
| 1. Absolutely not | 1 | 5.3 % |
| 1. I do not think so | 3 | 15.8 % |
| 1. I think so | 11 | 57.9 % |
| 1. Absolutely | 4 | 21.1 % |
| **5. How satisfied are you with the extent of help you received from the KisoMind_App_?** | 2.42 (0.69) | 10 (52.60%) |
| 1. Quite unsatisfied | 2 | 10.5 % |
| 1. Rather unsatisfied | 7 | 36.8 % |
| 1. Mostly satisfied | 10 | 52.6 % |
| 1. Very satisfied | 0 | 0 % |
| **6. Did the KisoMind_App_ help you to better cope with your problems?** | 2.74 (0.45) | 14 (73.70%) |
| 1. No, it made it worse | 0 | 0 % |
| 1. No, it did not really help | 0 | 0 % |
| 1. Yes, it helped a bit | 5 | 26.3 % |
| 1. Yes, it helped a lot | 14 | 73.7 % |
| **7. How satisfied are you with the KisoMind_App_ overall?** | 2.58 (0.769) | 12 (63.20%) |
| 1. Quite unsatisfied | 2 | 10.5 % |
| 1. Rather unsatisfied | 5 | 26.3 % |
| 1. Mostly satisfied | 11 | 57.9 % |
| 1. Very satisfied | 1 | 5.3 % |
| **8. Would you use the KisoMind_App_ again?** | 2.95 (0.84) | 14 (73.70%) |
| 1. Absolutely not | 1 | 5.3 % |
| 1. I do not think so | 4 | 21.1 % |
| 1. I think so | 9 | 47.4 % |
| 1. Yes | 5 | 26.3 % |

*Notes.* M = mean, SD = standard deviation, n = sample, % = percentage.

**Table S4**

# *Detailed analysis of the system usability scale (SUS)*

| Item | *M* or *n* | *SD* or % |
| --- | --- | --- |
| **System Usability Scale** |  |  |
| I think I would use the app on a regular basis. | 3.74 | 1.14 |
| 1. Completely disagree | 0 | 0 % |
| 1. Somewhat disagree | 3 | 15.8 % |
| 1. Neutral | 6 | 31.6 % |
| 1. Somewhat agree | 3 | 15.8 % |
| 1. Completely agree | 6 | 36.8 % |
| The app seems needlessly complicated. | 1.47 | 0.77 |
| 1. Completely disagree | 13 | 68.4 % |
| 1. Somewhat disagree | 3 | 15.8 % |
| 1. Neutral | 3 | 15.8 % |
| 1. Somewhat agree | 0 | 0 % |
| 1. Completely agree | 0 | 0 % |
| I thought that the app was easy to use. | 4.74 | 0.73 |
| 1. Completely disagree | 0 | 0 % |
| 1. Somewhat disagree | 1 | 5.3 % |
| 1. Neutral | 0 | 0 % |
| 1. Somewhat agree | 2 | 10.5 % |
| 1. Completely agree | 16 | 84.2 % |
| I think I would need technical support to use the app properly. | 1.79 | 0.97 |
| 1. Completely disagree | 9 | 47.4 % |
| 1. Somewhat disagree | 7 | 36.8 % |
| 1. Neutral | 1 | 5.3 % |
| 1. Somewhat agree | 2 | 10.5 % |
| 1. Completely agree | 0 | 0 % |
| I thought that the different functions of the app were well integrated. | 3.74 | 1.01 |
| 1. Completely disagree | 0 | 0 % |
| 1. Somewhat disagree | 3 | 15.8 % |
| 1. Neutral | 2 | 10.5 % |
| 1. Somewhat agree | 9 | 47.4 % |
| 1. Completely agree | 5 | 26.3 % |
| The app seems too inconsistent. | 2.11 | 0.99 |
| 1. Completely disagree | 7 | 36.8 % |
| 1. Somewhat disagree | 4 | 21.1 % |
| 1. Neutral | 7 | 36.8 % |
| 1. Somewhat agree | 1 | 5.3 % |
| 1. Completely agree | 0 | 0 % |
| I think that most people could learn to use the app very quickly. | 4.74 | 0.45 |
| 1. Completely disagree | 0 | 0 % |
| 1. Somewhat disagree | 0 | 0 % |
| 1. Neutral | 0 | 0 % |
| 1. Somewhat agree | 5 | 26.3 % |
| 1. Completely agree | 14 | 73.7 % |
| The app seems too difficult to use | 1.37 | 0.59 |
| 1. Completely disagree | 13 | 68.4 % |
| **Table S4** (*continued*) |  |  |
| Item | *M* or *n* | *SD* or % |
| 1. Somewhat disagree | 5 | 26.3 % |
| 1. Neutral | 1 | 5.3 % |
| 1. Somewhat agree | 0 | 0 % |
| 1. Completely agree | 0 | 0 % |
| I feel very safe in using the app. | 4.37 | 0.68 |
| 1. Completely disagree | 0 | 0 % |
| 1. Somewhat disagree | 0 | 0 % |
| 1. Neutral | 2 | 10.5 % |
| 1. Somewhat agree | 8 | 42.1 % |
| 1. Completely agree | 9 | 47.4 % |
| I had to learn a lot to properly use the app. | 1.74 | 1.14 |
| 1. Completely disagree | 12 | 63.2 % |
| 1. Somewhat disagree | 3 | 15.8 % |
| 1. Neutral | 1 | 5.3 % |
| 1. Somewhat agree | 3 | 15.8 % |
| 1. Completely agree | 0 | 0 % |

*Notes.* M = mean, SD = standard deviation, n = sample, % = percentage.

**Table S5**

# *Self-developed questionnaire to assess detailed feedback about the KISOMind_app_*

| Item | *M* or *n* | *SD* or % |
| --- | --- | --- |
| **App usefulness rated after the testing period** | | |
| Please rate the usefulness of the introduction into the app. | 4.05 | 0.84 |
| Not useful at all | 0 | 0 % |
| Rather not useful | 0 | 0 % |
| Neutral | 6 | 31.6 % |
| Rather useful | 6 | 31.6 % |
| Very useful | 7 | 36.8 % |
| Please rate the usefulness of the medication planner. | 3.89 | 1.10 |
| Not useful at all | 0 | 0 % |
| Rather not useful | 3 | 15.8 % |
| Neutral | 3 | 15.8 % |
| Rather useful | 6 | 31.6 % |
| Very useful | 7 | 36.8 % |
| Please rate the usefulness of the calendar. | 3.11 | 0.87 |
| Not useful at all | 0 | 0 % |
| Rather not useful | 5 | 26.3 % |
| Neutral | 8 | 42.1 % |
| Rather useful | 5 | 26.3 % |
| Very useful | 1 | 5.3 % |
| Please rate the usefulness of the mood check. | 4.32 | 1.10 |
| Not useful at all | 1 | 5.3 % |
| Rather not useful | 1 | 5.3 % |
| Neutral | 0 | 0 % |
| Rather useful | 6 | 31.6 % |
| Very useful | 11 | 57.9 % |
| Please rate the ‘advice-of-the-day’. | 4.00 | 1.00 |
| Not useful at all | 0 | 0 % |
| Rather not useful | 1 | 5.3 % |
| Neutral | 6 | 31.6 % |
| Rather useful | 4 | 21.1 % |
| Very useful | 8 | 42.1 % |
| Please rate the usefulness of the app in general. | 3.68 | 1.10 |
| Not useful at all | 0 | 0 % |
| Rather not useful | 4 | 21.1 % |
| Neutral | 3 | 15.8 % |
| Rather useful | 7 | 36.8 % |
| Very useful | 5 | 26.3 % |
| **App Understandability Rated After the Testing Period** | | |
| Please rate the understandability of the introduction into the app. | 4.47 | 0.69 |
| Not understandable at all | 0 | 0 % |
| Rather not understandable | 0 | 0 % |
| Neutral | 2 | 10.5 % |
| Rather understandable | 6 | 31.6 % |
| Very understandable | 11 | 57.9 % |
| Please rate the understandability of the medication planner. | 4.32 | 0.88 |
| Not understandable at all | 0 | 0 % |
|  |  |  |
| **Table S5** (*continued*) |  |  |
| Item | *M* or *n* | *SD* or % |
| Rather not understandable | 1 | 5.3 % |
| Neutral | 2 | 10.5 % |
| Rather understandable | 6 | 31.6 % |
| Very understandable | 10 | 52.6 % |
| Please rate the understandability of the calendar. | 3.42 | 1.21 |
| Not understandable at all | 0 | 0 % |
| Rather not understandable | 6 | 31.6 % |
| Neutral | 4 | 21.1 % |
| Rather understandable | 4 | 21.1 % |
| Very understandable | 5 | 26.3 % |
| Please rate the understandability of the mood check. | 4.58 | 0.50 |
| Not understandable at all | 0 | 0 % |
| Rather not understandable | 0 | 0 % |
| Neutral | 0 | 0 % |
| Rather understandable | 8 | 42.1 % |
| Very understandable | 10 | 57.9 % |
| Please rate the understandability of the ‘advice-of-the-day’. | 4.26 | 0.73 |
| Not understandable at all | 0 | 0 % |
| Rather not understandable | 0 | 0 % |
| Neutral | 3 | 15.8 % |
| Rather understandable | 8 | 42.1 % |
| Very understandable | 8 | 42.1 % |
| Please rate the understandability of the app in general. | 4.37 | 0.59 |
| Not understandable at all | 0 | 0 % |
| Rather not understandable | 0 | 0 % |
| Neutral | 1 | 5.3 % |
| Rather understandable | 10 | 52.6 % |
| Very understandable | 8 | 42.1 % |
| **App Usability Rated After the Testing Period** |  |  |
| Please rate the usability of the medication planner. | 3.95 | 1.17 |
| Not easy to use at all | 1 | 5.3 % |
| Rather not easy to use | 2 | 10.5 % |
| Neutral | 1 | 5.3 % |
| Rather easy to use | 8 | 42.1 % |
| Very easy to use | 7 | 36.8 % |
| Please rate the usability of the calendar. | 3.42 | 1.17 |
| Not easy to use at all | 0 | 0 % |
| Rather not easy to use | 6 | 31.6 % |
| Neutral | 3 | 15.8 % |
| Rather easy to use | 6 | 31.6 % |
| Very easy to use | 4 | 21.1 % |
| Please rate the usability of the mood check. | 4.26 | 1.04 |
| Not easy to use at all | 0 | 0 % |
| Rather not easy to use | 2 | 10.5 % |
| Neutral | 2 | 10.5 % |
| Rather easy to use | 4 | 21.1 % |
| Very easy to use | 11 | 57.9 % |
| Please rate the usability of the app in general. | 4.21 | 0.97 |
|  |  |  |
| **Table S5** (*continued*) |  |  |
| Item | *M* or *n* | *SD* or % |
| Not easy to use at all | 0 | 0 % |
| Rather not easy to use | 2 | 10.5 % |
| Neutral | 1 | 5.3 % |
| Rather easy to use | 7 | 36.8 % |
| Very easy to use | 9 | 47.4 % |
| **App Usage Rated After the Testing Period** |  |  |
| Please rate how often the medication planner successfully reminded you of taking your medication. | 3.68 | 1.73 |
| Not at all | 5 | 26.3 % |
| Less than once per week | 0 | 0 % |
| Once per week | 1 | 5.3 % |
| Multiple times per week | 3 | 15.8 % |
| Daily | 10 | 52.6 % |
| Please rate how often you used the calendar. | 2.53 | 1.38 |
| Not at all | 6 | 31.6 % |
| Less than once per week | 4 | 21.1 % |
| Once per week | 4 | 21.1 % |
| Multiple times per week | 3 | 15.8 % |
| Daily | 2 | 10.5 % |
| Please rate how often you answered the mood check. | 4.32 | 0.67 |
| Not at all | 0 | 0 % |
| Less than once per week | 0 | 0 % |
| Once per week | 2 | 10.5 % |
| Multiple times per week | 9 | 47.4 % |
| Daily | 8 | 42.1 % |
| When you answered the mood check, how many questions did you answer per day? | 4.79 | 0.91 |
| 1 out of 3 | 1 | 5.3 % |
| 2 out of 3 | 0 | 0 % |
| 3 out of 3 | 18 | 94.7 % |
| Please rate how often you read the ‘advice-of-the-day’. | 3.68 | 1.33 |
| Not at all | 2 | 10.5 % |
| Less than once per week | 2 | 10.5 % |
| Once per week | 2 | 10.5 % |
| Multiple times per week | 7 | 36.8 % |
| Daily | 6 | 31.6 % |
| Please rate how often you used the app in general. | 4.47 | 0.69 |
| Not at all | 0 | 0 % |
| Less than once per week | 0 | 0 % |
| Once per week | 2 | 10.5 % |
| Multiple times per week | 6 | 31.6 % |
| Daily | 11 | 57.9 % |
| The ‘advice-of-the-day’ was… | 4.11 | 0.80 |
| Not interesting at all | 0 | 0 % |
| Rather not interesting | 0 | 0 % |
| Neutral | 5 | 26.3 % |
| Rather interesting | 7 | 36.8 % |
| Very Interesting | 7 | 36.8 % |
| The ‘advice-of-the-day’ was… | 3.84 | 0.76 |
|  |  |  |
| **Table S5** (*continued*) |  |  |
| Item | *M* or *n* | *SD* or % |
| Not entertaining at all | 0 | 0 % |
| Rather not entertaining | 0 | 0 % |
| Neutral | 7 | 36.8 % |
| Rather entertaining | 8 | 42.1 % |
| Not entertaining at all | 4 | 21.1 % |
| **App Design Rated After the Testing Period** |  |  |
| Please rate the design of the medication planner. | 3.74 | 0.93 |
| Not appealing at all | 0 | 0 % |
| Rather not appealing | 3 | 15.8 % |
| Neutral | 2 | 10.5 % |
| Rather appealing | 11 | 57.9 % |
| Very appealing | 3 | 15.8 % |
| Please rate the design of the calendar. | 3.42 | 1.07 |
| Not appealing at all | 0 | 0 % |
| Rather not appealing | 5 | 26.3 % |
| Neutral | 4 | 21.1 % |
| Rather appealing | 7 | 36.8 % |
| Very appealing | 3 | 15.8 % |
| Please rate the design of the mood check. | 4.42 | 0.90 |
| Not appealing at all | 0 | 0 % |
| Rather not appealing | 1 | 5.3 % |
| Neutral | 2 | 10.5 % |
| Rather appealing | 4 | 21.1 % |
| Very appealing | 12 | 63.2 % |
| Please rate the design of the ‘advice-of-the-day’. | 3.89 | 0.93 |
| Not appealing at all | 0 | 0 % |
| Rather not appealing | 2 | 10.5 % |
| Neutral | 3 | 15.8 % |
| Rather appealing | 9 | 47.4 % |
| Very appealing | 5 | 26.3 % |
| Please rate the design of the app in general. | 3.79 | 0.91 |
| Not appealing at all | 0 | 0 % |
| Rather not appealing | 2 | 10.5 % |
| Neutral | 4 | 21.1 % |
| Rather appealing | 9 | 47.4 % |
| Very appealing | 4 | 21.1 % |
| **App-related Influences Rated After the Testing Period** |  |  |
| The medication planner has… | 3.58 | 1.42 |
| Not helped me in correctly taking my medication | 3 | 15.8 % |
| Rather not helped me in correctly taking my medication | 1 | 5.3 % |
| Neutral | 3 | 15.8 % |
| Rather helped me in correctly taking my medication | 6 | 31.6 % |
| Really helped me in correctly taking my medication | 6 | 31.6 % |
| The calendar has… | 2.74 | 0.93 |
| Not helped me in planning my day or important therapeutic appointments | 1 | 5.3 % |
| Rather not helped me in planning my day or important therapeutic appointments | 7 | 36.8 % |
|  |  |  |
| **Table S5** (*continued*) |  |  |
| Item | *M* or *n* | *SD* or % |
| Neutral | 8 | 42.1 % |
| Rather helped me in planning my day or important therapeutic appointments | 2 | 10.5 % |
| Really helped me in planning my day or important therapeutic appointments | 1 | 5.3 % |
| The ‘advice-of-the-day’ has… | 3.79 | 1.08 |
| Not made me open the app more often | 1 | 5.3 % |
| Rather not made me open the app more often | 1 | 5.3 % |
| Neutral | 4 | 21.1 % |
| Rather made me open the app more often | 8 | 42.1 % |
| Really made me open the app more often | 5 | 26.3 % |
| The mood check would… | 3.74 | 1.09 |
| Not be helpful at all in recognizing early warning signs of a relapse | 1 | 5.3 % |
| Rather not be helpful in recognizing early warning signs of a relapse | 2 | 10.5 % |
| Neutral | 2 | 10.5 % |
| Rather be helpful in recognizing early warning signs of a relapse | 10 | 52.6 % |
| Really be helpful in recognizing early warning signs of a relapse | 4 | 21.1 % |
| The mood check would… | 3.79 | 1.13 |
| Not be helpful at all in improving self-efficacy | 1 | 5.3 % |
| Rather not be helpful in improving self-efficacy | 1 | 5.3 % |
| Neutral | 5 | 26.3 % |
| Rather be helpful in improving self-efficacy | 6 | 31.6 % |
| Really be helpful in improving self-efficacy | 6 | 31.6 % |
| If you saw that your mood is steadily declining over a longer period of time by looking at the mood check in the KISO-app, how would that influence your motivation to use the app?  It would… | 3.79 | 1.03 |
| Demotivate me to use the app | 1 | 5.3 % |
| Rather demotivate me to use the app | 0 | 0 % |
| Neutral | 6 | 31.6 % |
| Rather not demotivate me to use the app | 7 | 36.8 % |
| Not demotivate me at all to use the app | 5 | 26.3 % |
| Have you ever taken your medication incorrectly because you entered it incorrectly into the app? | 2.0 | 0.0 |
| Yes | 0 | 0 % |
| No | 19 | 100 % |
| Have you ever taken your medication incorrectly because the app provided you with wrong information, due to a technical error? | 1.95 | 0.22 |
| Yes | 1 | 5.3 % |
| No | 18 | 94.7 % |

*Notes.* M = mean, SD = standard deviation, n = sample, % = percentage. The scale used for the self-created questionnaire ranges from 1 to 5, with higher scores indicating a higher level of the concept in question. Item 36 and 37 are rated on a binary (Yes/No) scale.

**Table S6.**

# *Baseline demographics and clinical data of all participants (N = 20)*

| Characteristics | *M* or *n* | *SD* or % |
| --- | --- | --- |
| **Sociodemographic data and diagnosis** |  |  |
| Age | 38.4 | 8.62 |
| Gender |  |  |
| Female | 7 | 35 |
| Male | 13 | 65 |
| Other | 0 | 0 |
| Primary Diagnosis |  |  |
| Schizophrenia | 18 | 78 |
| Schizoaffective Disorder | 2 | 9 |
| Comorbid Diagnoses |  |  |
| Depression | 3 | 13 |
| Anxiety Disorder | 1 | 1 |
| **Clinical symptoms** |  |  |
| PANSS |  |  |
| Positive scale | 14.1 | 5.81 |
| Negative scale | 16.8 | 7.12 |
| Total score | 30.9 | 10.42 |
| DASS |  |  |
| Depression scale | 9.30 | 7.51 |
| Anxiety scale | 8.4 | 3.92 |
| Stress scale | 11.70 | 9.27 |
| Total score | 29.40 | 18.46 |

*Notes.* M = mean, SD = standard deviation, n = sample, % = percentage, DASS = Depression Anxiety Stress Scale, m = mean, SD = standard deviation.
